# Multiple Rare Anatomic Variations in Anterior and Posterior Cerebral Circulation: A Case Report

**DOI:** 10.7759/cureus.59236

**Published:** 2024-04-28

**Authors:** Mugurel C Rusu, Mihai Lazăr, Alexandra D Vrapciu, Aida Geamănu

**Affiliations:** 1 Division of Anatomy, Faculty of Dentistry, “Carol Davila” University of Medicine and Pharmacy, Bucharest, ROU; 2 Division of Physiopathology 2, Faculty of Medicine, “Carol Davila” University of Medicine and Pharmacy, Bucharest, ROU; 3 Division of Ophthalmology, University Emergency Hospital, Faculty of Medicine, “Carol Davila” University of Medicine and Pharmacy, Bucharest, ROU

**Keywords:** posterior cranial fossa, cerebellum, anterior cerebral artery, pica, primitive lateral basilovertebral anastomosis

## Abstract

Anatomic variations of intracranial arteries are of paramount importance in neurosurgery and interventional radiology. Three extremely rare arterial variants were found by observing the intracranial vascular anatomy on the magnetic resonance angiography files of a 56-year-old female patient. Firstly, on the left side of the vertebrobasilar axis, a persistent primitive lateral basilovertebral anastomosis was found uniting the left anterior inferior and posterior inferior cerebellar arteries; further, the left anterior inferior cerebellar artery looped above the nerves of the internal auditory canal. Secondly, the right posterior inferior cerebellar artery was shown to be leaving the vertebral artery and had a distal fenestration of the telovelotonsillar segment. Such cases of distal fenestrated posterior inferior cerebellar artery are rare. Thirdly, a partly duplicated anterior communicating artery was also found in the anterior circulation. In conclusion, magnetic resonance angiography helps distinguish and detail discrete and delicate rare arterial variants.

## Introduction

The vertebral arteries (VAs) unite in the posterior fossa to form the basilar artery (BA), and cerebellar arteries emerge from the vertebrobasilar system. The posterior inferior cerebellar artery (PICA) leaves the VA, has a proximal anterior medullary segment, and continues with successive lateral medullary, tonsillomedullary, telovelotonsillar, and cortical segments [1]. The telovelotonsillar segment forms a cranially convex loop. Fenestrations of PICA were rarely reported [2-5]. The persisting primitive lateral basilovertebral anastomosis (PLBA), or Padget's anastomosis, occasionally is parallel to the VA and the BA and unites in different morphological patterns, either successive cerebellar arteries or a cerebellar artery with the vertebrobasilar trunk [6]. Gregg and Gailloud (2017) reported such different patterns of PLBA [6]. On the other hand, in the anterior cerebral circulation, the anterior communicating artery (AComA) unites the anterior cerebral arteries (ACAs) and could appear fenestrated [7].

Multiple rare intracranial arterial variants encountered in one case are reported herein: a PLBA, PICA’s distal fenestration, and the partial duplication of the AComA.

## Case presentation

The archived magnetic resonance (MR) angiogram of a 56-year-old female patient was studied anatomically. The MRI files were anonymously studied retrospectively, and the clinical details remained unknown. The MR examination was performed on a 3T Magnetom Vida system (Siemens Healthineers, Erlangen, Germany) using a Head Neck 20TCS coil. The scan protocol included following sequences: t1_fl2d_sag_4mm, t2_tse_tra_512_4mm, t2_tse_dark-fluid_tra_3mm, resolve_4scan_trace_tra_p2_192_4mm, t2_swi_tra_p2_2mm, t1_fl2d_tra_4mm, t2_tse_dark-fluid-cor_3mm, 3d_tof, t1_mprage_tra_p2_iso_0.9mm. We injected a volume of 20mL Clariscan followed by 20mL saline bolus with a flow of 3mL/sec and repeated t1_mprage_tra_p2_iso_0.9mm (with automatic subtraction of the non-enhanced sequence), followed by t1_se_tra_blood-suppr._MTC_4mm. Multiplanar and volumetric reconstruction were performed using the t1_mprage sequence. The anatomic variants were documented using the Horos 3.3.6 software (Horos Project, Annapolis, MD, USA) on planar slices and three-dimensional volume renderings. Measurements were done on planar slices. For three-dimensional volume renderings, the 16-bit CLUT editor was used. The research followed principles from the Code of Ethics of the World Medical Association (Declaration of Helsinki). The Ethics Committee of the University Emergency Hospital, Bucharest approved the study (approval no. 10540/16.02.2022).

**Figure 1:**
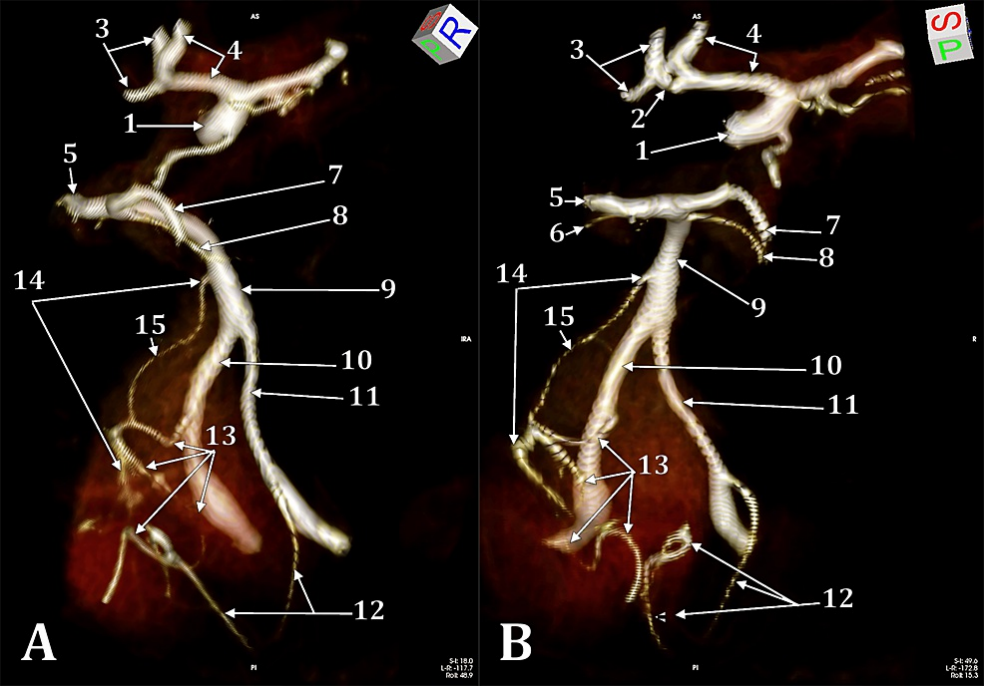
Three-dimensional volume renderings of persisting longitudinal basilovertebral anastomosis. Right postero-infero-lateral view (A). Posterior view (B). (1) right internal carotid artery; (2) partly duplicated anterior communicating artery; (3) left anterior cerebral artery; (4) right anterior cerebral artery; (5) left posterior cerebral artery; (6) left superior cerebellar artery; (7) right posterior cerebral artery; (8) right superior cerebellar artery; (9) basilar artery; (10) left vertebral artery; (11) right vertebral artery; (12) right posterior inferior cerebellar artery; (13) left posterior inferior cerebellar artery; (14) left anterior inferior cerebellar artery; (15) left persisting longitudinal basilovertebral anastomosis.

The BA formed on clivus from the union of the VAs. It had a median course and, from its rostral end, left the superior cerebellar and posterior cerebral arteries. The BA was 2.87 cm long. The left PICA origin from the antero-medial side of the left VA was 0.94 cm proximal to the BA origin. The left anterior inferior cerebellar artery (AICA) origin was from the left side of the BA, at 0.85 cm distal to its origin. The left PICA and AICA were united by a 1.2-mm-calibre longitudinal anastomosis located on the left side of the left VA and BA. This was, therefore, considered a persistent primitive lateral basilovertebral anastomosis (PLBVA) (Figure 1). Distal to that anastomosis, the AICA continued looping on the nerves of the internal auditory canal towards its cerebellar territory.

**Figure 2:**
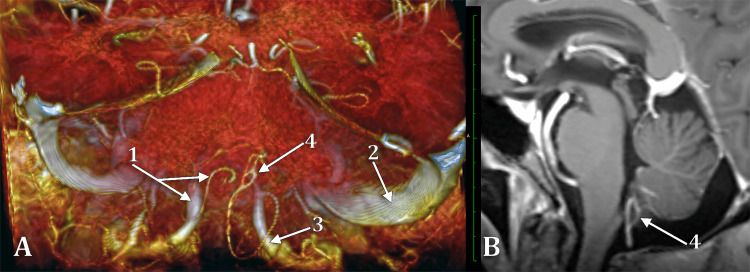
Distal (telovelotonsillar) fenestration of the right posterior inferior cerebellar artery. (A) Three-dimensional volume rendering, postero-superior view of the posterior cranial fossa. (B) Sagittal slice through the telovelotonsillar segment of the right posterior inferior cerebellar artery. (1) left vertebral and posterior inferior cerebellar arteries; (2) right sigmoid sinus; (3) right vertebral artery; (4) fenestrated telovelotonsillar segment of the right posterior inferior cerebellar artery.

The right PICA left the right VA at 1.61 cm distally to VAs dural ring. Then, it crossed the VA posteriorly and descended in the cerebellomedullary cistern. Then, it modified its course around the right flocculonodular lobe and ascended dorsally to the inferior medullary velum, where it presented a longitudinal fenestration of 0.30/0.14 cm on the cranial loop of that PICA (Figure 2). It further had bilateral cerebellar distribution.

In the anterior part of Willis's circle, a variant of the anterior communicating complex was found: a 1.2/1.3 mm partly duplicated AComA with two arms inserted into the right ACA and a single arm into the left ACA (Figure 1).

## Discussion

**Figure 3:**
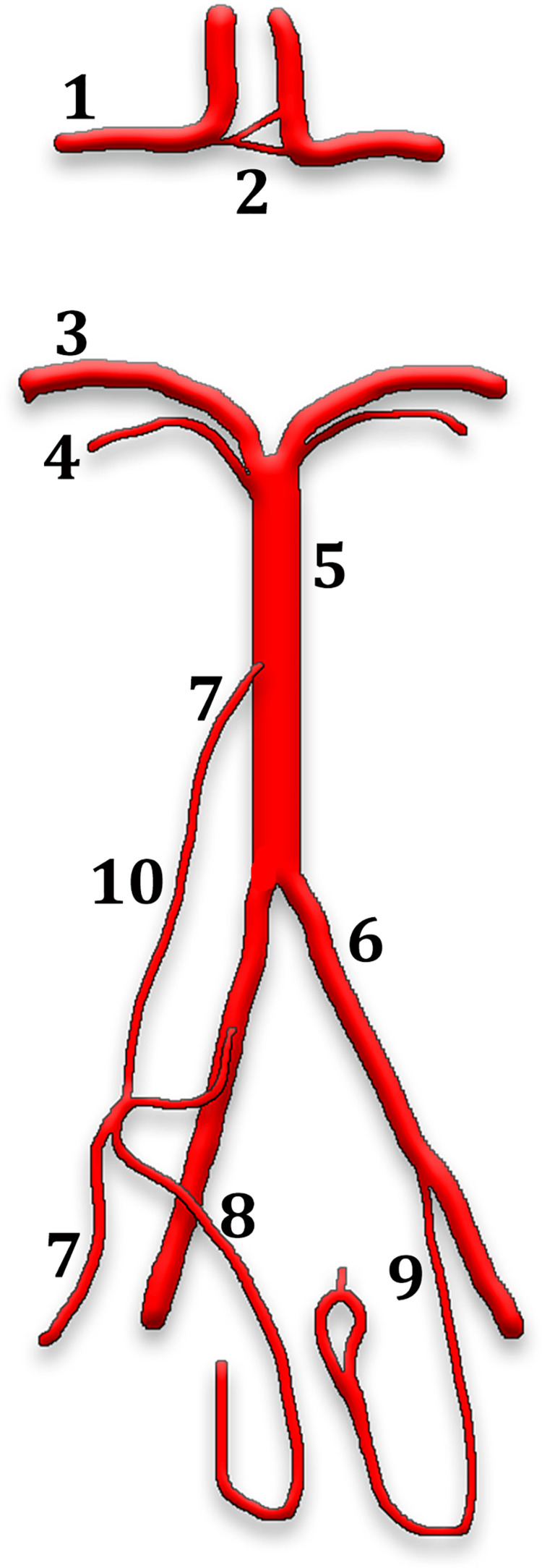
Schematic diagram of the reported variants (dorsal view). (1) anterior cerebral artery; (2) partly duplicated anterior communicating artery; (3) posterior cerebral artery; (4) superior cerebellar artery; (5) basilar artery; (6) vertebral artery; (7) left anterior inferior cerebellar artery; (8) left posterior inferior cerebellar artery; (9) right posterior inferior cerebellar artery; (10) persisting longitudinal primitive basilovertebral anastomosis. This illustration has been created by the corresponding author.

The anatomical pattern of the PLBA we report here (Figure 3) is not superposable compared to any of those reported by Gregg and Gailloud (2017) [6]. Pedro et al. (2021) also reported a PLBA variant that joined the PICA with the junction of the VA and BA, the AICA on that side being absent [8]. The variant of Pedro et al. (2021) is almost identical to one of the variants of Gregg and Gailloud (2017), but in which the AICA was present [6,8]. Also, Ota et al. (2022) reported a case with a PLBA joining the AICA and the origin of the PICA on that side [9]. In all morphological variants reported previously [6,8,9], the PLBA appeared as an additional arterial trunk paralleling the BA and VA.

Before VA formation, the posterior circulation is fed by a series of transverse anastomoses connecting the carotid circulation to the longitudinal neural arteries (LNAs) [6]. The LNAs subsequently form the basilar artery (BA) [6]. Among the presegmental transverse anastomoses is the proatlantal artery (ProA) of Padget-Arey, which Padget (1954) termed the "suboccipital artery" [6,10]. The ProA participates in forming the V3 and V4 segments of the VA [6]. The ProA provides continuity between the BA and the cervical VA in adults [6]. The PLBA is a longitudinal anastomotic canal that temporarily connects the developing branches of the vertebrobasilar system [6]. The PLBA results from the dorsal branch of the ProA; it travels along the lateral side of the LNA, to which it connects via several transverse anastomoses [6]. Some of these anastomoses will later become the cerebellar arteries' proximal segments and other BA branches [6]. Padget anticipated in 1954 that remnant segments of the PLBA could result in anatomic variations of the adult vertebrobasilar system in the posterior cranial fossa [6,10].

Fenestration of the PICA is exceedingly rare [3]. Theodosopoulos and Lawton first reported a fenestrated PICA in 2000 [5]. Further, Lesley (2008) reported a left-sided, fenestrated, proximal anterior medullary segment of PICA [3].

Lee et al. (2009) reported a proximal anterior medullary fenestration of a PICA with double origin [11]. Cho et al. (2011) also reported the double origin of PICA, with juxta-proximal fenestration of the caudal component [1]. The authors adequately described the geometry of those variants at that time. However, the cranial component evidence in those reports [11,12] appears instead as a PLBA and should be added to the PLBA variants, which were nicely described later by other authors [6,8,9].

Kumar et al. (2012) reported fenestrations of the right PICA and vertebrobasilar junction in a case with a VA aneurysm [2]. In that case, the PICA fenestration was on the proximal anterior medullary segment [2].

Maeda et al. (2017) documented previous reports [2,3,5] of fenestrated PICAs, counting that their report is just the fourth one [4]. This makes the present report the fifth evidence of a fenestrated PICA. They regarded the variant of Cho et al. (2011) as PICA’s duplication [4]. This seems fitting, as the arms of the large fenestrations Cho et al. presented had distinctive insertions into the respective VA [12]. Maeda observed that in the previous three cases of fenestrated PICAs [2,3,5] the fenestrations were on the proximal anterior medullary segment. Maeda found the VA continued as PICA and a fenestration on the cranial loop of that PICA, with an aneurysm on that fenestration [4]. We also found a fenestrated cranial loop of PICA on the right side, but PICA left a complete V4 segment of the VA in our case. Our finding supports the previous research of Maeda. By correlating previous evidence [2-5,11,12], two topographical types of PICA could be defined: type 1, the proximal anterior medullary fenestrations; and type 2, the distal telovelotonsillar fenestrations. However, fenestrations of the intracranial arteries are rare anomalies, considered to result from the incomplete fusion of primitive embryologic vessels [13]. As the cerebellar arteries divide and form networks as they advance towards their specific territory [14], a persisting gap of such a primitive network would appear as a fenestrated segment of that artery in adults.

Variations of the ACA have rarely been studied by MR angiography [15,16] as was investigated in the present case. However, in almost 900 patients documented by MR angiography, there were no reported fenestrations or duplications of AComA [15]. Fenestrations of AComA were found in 12/227 cases (5.3%) by de Gast et al. (2008) [17]. These authors described "a wide variety in fenestrations of the AComA from simple duplication to stepladder-like configurations" without distinguishing between fenestrations and duplications. Later, duplications of AComA were classified by Uchino (2022) as complete (two parallel arms uniting the ACAs), partial (the two arms insert distinctively into one ACA and by a common trunk into the opposite one), and double partial (the two AComAs draw a lying X between the ACAs) [18]. AComA duplication is estimated at a 4.3% pooled prevalence [7]. Therefore, fenestrations and duplications of the AComA are rare, but specialists should be aware of such morphological possibilities when approaching the ACAs and AComA. The AComA develops from a vascular network that unites to a variable degree by the time of birth [17,19]. Therefore, the lack of fusion of such primitive networks would lead to fenestrations. Fenestrations do not predispose patients to aneurysms or vascular malformations remote from the fenestration site [13].

## Conclusions

The present report contributes a new PLBA pattern, distinguishes two types of PICA fenestration, anterior medullary and telovelotonsillar, and presents a rare variant of partly duplicated AComA. These variations could be remnants of the primitive vascular networks that failed to fuse or disappear before birth. Neurosurgeons should be aware of rare anatomical variations that could be documented carefully on MRI scans. 
